# The paradox of protection: rethinking challenge, wellbeing, and development in high-performance sport

**DOI:** 10.3389/fspor.2026.1965164

**Published:** 2026-10-07

**Authors:** Andrea Buchanan, Dave Collins, Urvi Khasnis

**Affiliations:** 1 Moray House School of Education and Sport, University of Edinburgh, Edinburgh, United Kingdom; 2 Grey Matters Performance Ltd., Stratford upon Avon, United Kingdom

**Keywords:** athlete wellbeing, challenge, high-performance sport, sport psychology, stress appraisal, talent development

## 1 Introduction: the growing emphasis of wellbeing in sport psychology

Stress and challenge are inevitable in high-performance sport. Athletes pursuing excellence routinely encounter uncertainty, failure, deselection, injury, competitive pressure, and emotionally demanding experiences. These experiences are rarely comfortable, yet are often central to learning, adaptation, and the development of expertise (1).

Over recent decades, sport psychology has undergone an important and largely welcome shift towards athlete wellbeing, mental health, and safeguarding. International consensus frameworks have rightly emphasised the need to recognise psychological distress, improve access to support, and protect athletes from unnecessary harm (2). This conversation has become increasingly sophisticated, recognising that athlete wellbeing is shaped by relationships, organisational cultures, and the broader performance environment (3, 4). These developments represent important progress.

However, has this shift come with an unintended cost?

We suggest that sport psychology has become more sophisticated in identifying the risks of excessive challenge than the developmental risks of insufficient challenge. Because harm is often immediate and visible, while the costs of under-challenge may emerge only later, policy and practice may default too readily towards reducing difficult demands. This risks treating discomfort as a problem to be alleviated rather than a potential feature of ambitious development.

Discomfort, distress, and harm are not equivalent. Some psychological unpleasantness may be developmentally desirable because athletes cannot develop capacities for uncertainty, difficult emotion, failure, or pressure without encountering those demands. The task is therefore to distinguish legitimate developmental challenge from avoidable harm, recognising that the balance between protection, support, and challenge will vary across athletes, circumstances, and stages of development. Accordingly, this paper examines challenge as a central mechanism of development, stress as a principal way challenge is psychologically experienced, and the implications of both for psychological support. We argue that sport psychology should keep athletes safe from harm without making them safe from the challenges through which ambitious development occurs.

## 2 Challenge as a mechanism of development

Progress in high-performance sport requires athletes to adapt when their existing capacities no longer meet emerging demands. Development rarely occurs through uninterrupted success; athletes encounter uncertainty, failure, deselection, injury, competitive pressure, and emotional disruption that require them to reconsider and refine how they perform (1). Here, development refers to an enduring expansion or reorganisation of psychological, behavioural, or technical capacities. Challenge creates the discrepancy between what an athlete can currently manage and what future performance requires. Without that discrepancy, there is little impetus for adaptation and limited opportunity for development (5).

Challenge is not the same as unnecessary adversity or avoidable harm. Exposure to difficulty does not guarantee growth, and subsequent success cannot justify inappropriate practice, even in hindsight. However, high-performance development necessarily involves demands that are uncomfortable and sometimes genuinely distressing. When such demands are proportionate, developmentally relevant, and accompanied by appropriate support and recovery, the disruption they create can prompt learning and adaptation (6).

Some forms of psychological unpleasantness may therefore be developmentally desirable, rather than merely unavoidable. Athletes cannot learn to tolerate uncertainty without experiencing it, regulate difficult emotions without encountering them, or develop the capacity to perform under pressure without practising under pressure (7). In these circumstances, discomfort is not simply an unwanted by-product of development; it forms part of the developmental stimulus because it provides the context in which the relevant capacities are exercised. As such, removing the discomfort may also remove the opportunity for those capacities to develop. Its developmental value lies in what the experience requires of the athlete and how that experience is structured and supported (6).

Intensity alone cannot determine whether a demand promotes development or becomes overwhelming. It also depends on how the athlete appraises the demand and the resources available to meet it (8). Athletes' experiences of challenge are shaped by their existing capacities, previous experiences, and access to appropriate support (9). Appraisal may also change through perceived control, relationships, and interaction with the wider performance environment (10). The same demand may therefore be manageable for one athlete, overwhelming for another, or experienced differently by the same athlete over time.

Challenge is consequently best understood as a dynamic relationship between the athlete, the demand, and the performance environment. Development depends not on removing difficulty, but on enabling athletes to engage with, adapt to, and make sense of demanding experiences in ways that expand their capacities.

## 3 Distinguishing developmental challenge from avoidable harm

Recognising challenge as a mechanism of development does not mean that all demanding experiences are beneficial. Distress refers here to the unpleasant psychological experience that may accompany demanding circumstances, whereas harm involves a significant adverse effect on an athlete's health, safety, dignity, functioning, or longer-term development. The two may overlap, but they are not synonymous: athletes may experience distress without harm, while harm may develop without immediate distress. Wellbeing therefore means more than the absence of discomfort; it concerns athletes' capacity to function, develop, and pursue meaningful goals without unacceptable costs (2). Safeguarding involves (or at least should) preventing practices and environments that threaten these conditions (11).

The boundary between developmental challenge and avoidable harm is not fixed. It depends on the purpose and proportionality of the demand, the athlete's appraisal, available resources and support, and the consequences over time. A legitimate challenge may become harmful when demands are excessive or accumulate without adequate recovery, when athletes experience persistent helplessness or impairment, or when exposure continues despite deteriorating health. Research on burnout similarly highlights the interaction between demands, recovery, motivation, and the performance environment, rather than treating stressful experiences as inherently harmful (12).

Power and agency also matter. Although athletes cannot choose every training demand, performance standard, or selection decision, they should understand the purpose of a demand, be able to express concerns, and be able to report inappropriate practice without fear of disproportionate consequences. How demands are imposed may matter as much as the demands themselves: controlling environments have been associated with psychological need thwarting (13) and athlete burnout (14). Younger athletes require greater caution, as their dependence on adults may limit their capacity to challenge demanding practices (15). Neither a coach's developmental intention nor an athlete's apparent acceptance is sufficient to establish that a practice is appropriate. Equally, temporary discomfort is not sufficient evidence of harm. It may require adjusting the intensity, timing, duration, or support around a challenge rather than removing it. However, when health, safety, dignity, functioning, or longer-term development are threatened, protection from harm takes priority.

## 4 Stress as the psychological experience of challenge

If challenge creates the conditions for development, stress is one of the main ways challenge is psychologically experienced. High-performance demands often generate stress because athletes pursue goals that matter deeply to them. Its presence does not therefore necessarily indicate dysfunction or that something has gone wrong. Indeed, for many it may be of long duration. The more important distinction is between the expected stress of engaging with meaningful demands and stress that becomes persistent, impairing, or harmful (2).

The significance of stress depends on how it is appraised and how it develops over time. Lazarus' (8) transactional model conceptualises stress as a dynamic relationship between the demands an individual encounters and their appraisal of the resources available to meet them. Apparently similar situations may therefore produce markedly different responses. One athlete may experience a selection decision, serious injury, or major competition as manageable and meaningful, while another may find it overwhelming. These responses may also change as demands accumulate, resources diminish, or capacities develop through experience.

Challenge and threat theory further supports this position. When athletes appraise their resources as sufficient to meet situational demands, they are more likely to experience a challenge state (16). In sport, challenge states have been associated with more adaptive physiological responses, attentional control, and performance under pressure, whereas threat becomes more likely when perceived demands exceed available resources (17). Neither response is a fixed property of the event or the athlete. Appraisal is continually shaped by previous experience, perceived control, relationships, and interaction with the performance environment (10).

Importantly, the aim cannot be to eliminate stress from the preparation for high-performance sport. Athletes are unlikely to develop the capacity to perform under pressure if training consistently protects them from experiencing it (7). Appropriate exposure allows athletes to practise interpreting, regulating, and responding to stress before encountering comparable demands in competition. Removing stress indiscriminately may therefore reduce immediate discomfort while also limiting preparation for future demands.

The relevant question is not whether stress is good or bad, but what it signifies for this athlete, in this context, at this point in their development. For practitioners, this shifts the focus from routinely reducing stress to assessing and monitoring it. Psychological support should intervene when stress becomes overwhelming, persistent, or impairing, while helping athletes engage constructively with the pressures through which high-level capacities are developed.

## 5 Supporting development without compromising wellbeing

How psychological support is understood may shape whether athletes and coaches regard it as relevant to high performance. In cultures where toughness is associated with competence, seeking support may be interpreted as evidence of vulnerability or an inability to cope. Athletes may also worry that addressing distress will diminish their motivation, competitive edge, or capacity to tolerate pressure. Alongside concerns about stigma, confidentiality, and career consequences, these perceptions may discourage help-seeking (18).

Such concerns may reflect uncertainty about the purpose of psychological support. If psychology is understood primarily as reducing discomfort, those who regard challenge as integral to performance may question its value. Psychological support should also help athletes engage effectively with demanding experiences by appraising challenges accurately and regulating difficult emotions (8), recovering appropriately, and making meaning from disruption (5). Practitioners can strengthen athlete agency and help coaches calibrate, explain, and support legitimate challenge (6). They should also monitor whether stress is becoming cumulative or impairing (19). Where there are signs of harm, maltreatment, or significant deterioration, protection and appropriate intervention take priority (11). Where challenge remains proportionate, support should help athletes remain engaged long enough for adaptation and learning to occur.

The aim is not to eliminate stress or encourage it indiscriminately, but to help athletes engage constructively with legitimate challenge while recognising when intervention is required. Wellbeing and development should be advanced together, although they need not receive identical emphasis in every situation or at every developmental stage. Wellbeing is not synonymous with comfort, and discomfort is not, by itself, evidence of harm.

## 6 Conclusion: rebalancing wellbeing and development

The growing emphasis on wellbeing and safeguarding has delivered important progress. However, it has also left the developmental significance of difficult and unpleasant experiences comparatively under-specified. High-performance development involves uncertainty, failure, pressure, vulnerability, and emotional disruption. Although these experiences are not inherently beneficial, discomfort and distress are not necessarily evidence of harm. Some forms of unpleasantness are developmentally desirable because they provide the conditions in which athletes exercise and extend the capacities required for ambitious performance.

Rebalancing wellbeing and development requires more than asserting that both matter. It requires recognising that they will not always receive equal emphasis and that the appropriate balance will vary across athletes, contexts, and stages of development. There is also a need to acknowledge and redress the “concept creep” which has seen many psychological constructs uncritically expanded to incorporate completely different or more moderate forms of the original (20). At times, protection and recovery should take priority. At other times, however, development may require athletes to encounter and work through demands that temporarily unsettle their wellbeing. Treating the reduction of discomfort as the default response risks protecting athletes from the very experiences high-performance sport requires them to be prepared for.

Sport psychology must therefore distinguish avoidable harm from legitimate developmental challenge by attending to the athlete, the demand, the performance environment, and the consequences that emerge over time. Protection from harm remains fundamental, but it should not become protection from difficulty. The future of sport psychology lies in keeping athletes safe from harm while preparing them for, rather than shielding them from, the challenges through which ambitious development occurs.
